# Long-term outcomes of two first-generation trabecular micro-bypass stents (iStent) with phacoemulsification in primary open-angle glaucoma: eight-year results

**DOI:** 10.1186/s40662-021-00263-1

**Published:** 2021-11-16

**Authors:** Ali Salimi, Harrison Watt, Paul Harasymowycz

**Affiliations:** 1 Department of Ophthalmology, Faculty of Medicine, McGill University, Montreal, QC Canada; 2 Montreal Glaucoma Institute, Montreal, QC Canada; 3 Faculty of Medicine, McGill University, Montreal, QC Canada; 4 Department of Ophthalmology, University of Montreal, 4135 de Rouen, Montreal, QC H1V1G5 Canada

**Keywords:** Minimally invasive glaucoma surgery, MIGS, Trabecular micro-bypass stent, iStent, Glaucoma, Cataract surgery, Long-term outcomes

## Abstract

**Background:**

The short- and medium-term outcomes of iStent have been extensively studied; however, only few studies have investigated its long-term outcomes. Here, we assessed the long-term efficacy and safety of two iStents with concomitant cataract surgery in glaucomatous eyes while also evaluating measures of disease stability using visual field and optical coherence tomography (OCT) of the optic nerve and the macula throughout 8 years of follow-up.

**Methods:**

This longitudinal, single-center consecutive case series included glaucomatous eyes that underwent implantation of two first-generation trabecular micro-bypass stents (iStent) with concomitant cataract surgery. Eight-year efficacy outcomes included mean intraocular pressure (IOP) and medications, as well as surgical success. Eight-year safety outcomes included best-corrected visual acuity (BCVA), visual field mean deviation (VF-MD), cup-to-disc ratio (CDR), retinal nerve fiber layer (RNFL) thickness, ganglion cell-inner plexiform layer (GC-IPL) thickness, and adverse events.

**Results:**

A total of 62 eyes with primary open-angle glaucoma (POAG) were included. At 8 years postoperative, IOP reduced by 26% from 19.2 ± 3.9 mmHg preoperatively to 14.2 ± 2.4 mmHg (*P* < 0.001), 91.1% of eyes achieved IOP ≤ 18 mmHg (vs. 51.6% preoperatively), 69.6% of eyes achieved IOP ≤ 15 mmHg (vs. 14.5% preoperatively), and 25% of eyes achieved IOP ≤ 12 mmHg (vs. 1.6% preoperatively). Medication use decreased by 17.9% from 2.8 ± 1.1 preoperatively to 2.3 ± 1.2 (*P* = 0.018). Surgical success was 90%, as six eyes underwent subsequent glaucoma surgeries. Safety measures of BCVA, CDR, RNFL thickness and GC-IPL thickness remained stable through 8 years postoperative. VF-MD remained stable until postoperative year 5 and subsequently progressed according to the natural history of glaucomatous disease.

**Conclusions:**

Implantation of two iStents with concomitant cataract surgery is an effective and safe treatment option for surgery-naïve POAG eyes, evidenced by significant IOP and medication reductions, reasonable surgical success, and favorable safety outcomes, throughout the 8-year follow-up. Our data additionally supports the efficacy of this combined procedure in stabilizing or slowing disease progression.

**Supplementary Information:**

The online version contains supplementary material available at 10.1186/s40662-021-00263-1.

## Background

Glaucoma is an irreversible, progressive optic neuropathy and a major cause of blindness worldwide [1]. The incidence of this degenerative condition is expected to rise with the increase in the aging population [2]. While we have yet to find a cure for glaucoma, multiple medical and surgical techniques have emerged to delay disease progression. Through different mechanisms of action, these procedures attempt to decrease intraocular pressure (IOP), the only modifiable risk factor for glaucoma identified to date [3]. Patients benefit from different management approaches depending on their demographics, medication compliance, and disease type and severity. For instance, medical therapy is often the preferred approach for early cases of glaucoma [1]; however, it bears limitations such as ocular surface damage, diurnal IOP variations, and dependence on patient compliance [4–6]. On the other extreme, advanced glaucoma cases are often managed via more invasive glaucoma surgeries, such as filtering surgeries. The efficacy of such procedures in reducing the IOP has been well established; however, there remain many concerns regarding the safety of these procedures [7–9].

Over the past decade, and with the advent of minimally invasive glaucoma surgeries (MIGSs), the field of glaucoma has witnessed an explosion of new surgical techniques and research examining their efficacy and safety [10, 11]. MIGSs are advantageous due to their superior safety compared to traditional incisional glaucoma surgeries, minimal disruption of conjunctival and scleral tissue, faster recovery time, and compatibility with concurrent cataract surgery, while also offering a modest efficacy [12]. The iStent trabecular micro-bypass was the first MIGS device to receive the US Food and Drug Association (FDA) approval for treatment of mild-to-moderate open-angle glaucoma [13]. iStent reduces the IOP by bypassing the dysfunctional trabecular meshwork, allowing the drainage of aqueous humor from the anterior chamber into Schlemm’s canal [14].

A wealth of research has examined the outcomes of the first- or second-generation trabecular micro-bypass stents (iStent or iStent inject) with or without concomitant cataract surgery in various glaucoma subtypes and severities [13–50]. Although these studies have typically supported the safety and efficacy of this device in glaucomatous eyes, the data have been often limited to short- and medium-term outcomes. Few studies have investigated the outcomes of iStent beyond 5 years postoperative [51, 52], among which only one assessed disease stability in terms of visual field measures [52], and none investigated the structural measures of disease stability such as retinal nerve fiber layer (RNFL) and ganglion cell-inner plexiform layer (GC-IPL) thickness. Therefore, the aim of this study was to evaluate the long-term safety and efficacy of two iStents with concomitant cataract surgery in glaucomatous eyes.

## Methods

### Participants and study design

This longitudinal, consecutive case series included eyes that underwent cataract extraction with concomitant implantation of two first-generation trabecular micro-bypass stents (CE-TMS) between 2009 and 2012. Inclusion criteria consisted of glaucomatous damage evidenced by RNFL imaging, ganglion cell analysis, or visual field testing; a coexisting cataract; the need for reduction of IOP or glaucoma medications; and the availability of 8 years of follow-up data. Eyes with nasal peripheral anterior synechiae or conditions inhibiting clear visualization of the nasal trabecular meshwork, elevated episcleral venous pressure, or chronic ocular inflammation were deemed ineligible for the surgery. The severity of cataract was assessed according to the Lens Opacities Classification System II [53], and the decision to perform a concomitant cataract surgery was made based on the patient’s subjective vision complaints, followed by a discussion regarding the risks and benefits of the procedure. To increase the power and generalizability of the study, no exclusion criteria were set based on the diagnosis or severity of glaucoma. Glaucoma severity was classified according to the Hodapp-Anderson-Parrish visual field criteria with visual field mean deviation (VF-MD) no worse than −6 dB classified as mild, between −6 and −12 dB as moderate, and −12 dB or worse as severe glaucoma [54]. All eyes underwent the surgery at a single ophthalmology center by one experienced glaucoma specialist.

All procedures were performed in accordance with the tenets of the Declaration of Helsinki. The study was approved by the ethics review board of the local hospital and was exempted from patient consent due to its retrospective nature.

### Surgical technique

The surgical technique has been described in detail elsewhere [15–18]. In short, two areas with the greatest degree of trabecular meshwork pigmentation or with evidence of focal blood reflux, separated by at least two clock hours, were preoperatively marked at the corneal limbus. Under sterile conditions, and after the instillation of topical anesthetic drops, a clear corneal incision was made temporally, through which the two iStent devices were advanced and inserted into the nasal trabecular meshwork, adjacent to the collector channels identified preoperatively. Subsequently, standard phacoemulsification was performed using the same corneal incision.

The standard postoperative regimen included one tablet of oral acetazolamide 500 mg on the first evening, topical moxifloxacin 0.5% (3 times a day for 1 week), topical nepafenac ophthalmic solution 0.1% (3 times a day for 1 month), and loteprednol etabonate 0.5% (4 times per day, tapered down every 4 days). Glaucoma medications were modified postoperatively on a case-by-case basis, as per the surgeon’s discretion, according to preoperative IOP, glaucoma severity, tolerance of the eye drops, and glaucoma drops used in the contralateral eye.

### Outcome measures and statistical analyses

Eligible participants were identified, and their relevant preoperative and postoperative clinical data were extracted, up to 8 years postoperative. Outcome measures were categorized into efficacy and safety measures. Efficacy measures included postoperative change in IOP and glaucoma medication use, as well as surgical success. Surgical success was based on four failure criteria: Criterion-A consisted of glaucoma reoperation due to inadequate IOP control or disease progression; Criterion-B included eyes in which the use of glaucoma medications increased at ≥ 2 visits; Criterion-C included those that underwent selective laser trabeculoplasty (SLT) postoperatively; Criterion-D included all eyes that failed according to the former three criteria and the earliest failure date was considered for those failing according to two or more criteria. Safety measures included postoperative change in best-corrected visual acuity (BCVA), VF-MD, and structural measures of disease stability such as cup-to-disc ratio (CDR), RNFL thickness, and GC-IPL thickness. Of note, the average GC-IPL thickness was measured as the distance between the outer boundary of the nerve fiber layer and the outer boundary of the inner plexiform layer, obtained from the macular cube scan, and analyzed by the built-in Cirrus HD-OCT software [55]. Intraoperative and postoperative adverse events were noted.

To minimize the potential effect of regression toward the mean on IOP, the mean of the last two preoperative IOP measurements was used to define the baseline IOP for eyes in which there were two preoperative IOP measurements within the month prior to surgery. For statistical analyses, BCVA scores were converted to the logarithm of the minimum angle of resolution (logMAR). IOP spike was defined as an increase in IOP of > 50% or > 10 mmHg, relative to the preoperative IOP [56, 57]. To limit the bias from postoperative conditions and to ensure that the postoperative outcomes of eyes can be associated with CE-TMS, postoperative data of eyes that underwent additional glaucoma surgeries were censored in the analysis of subsequent follow-up time points.

Postoperative change in continuous variables of interest was assessed using the generalized estimating equations (GEE), a modelling approach that is free from distributional assumptions, and robust in the face of missing data of longitudinal studies [58]. GEE models were corrected for inter-eye correlation. Little's Test of Missing Completely at Random (MCAR) tested whether the missing data are missing completely at random. For completeness of analyses, we additionally performed multiple imputations using logistic regression models with 50 iterations and repeated the GEE models, as supplementary analyses. Proportional analyses were performed to assess the percentage of eyes with IOP ≤ 18 mmHg,  ≤ 15 mmHg,  ≤ 12 mmHg, as well as those that were medication-free, maintained or decreased their glaucoma medication use, or decreased their use by at least one medication. Kaplan–Meier curve assessed the cumulative 8-year survival according to the four failure criteria described above. All statistical analyses were performed using SPSS 26.0 (IBM, NY, USA) with alpha set at 0.05 for statistical significance.

## Results

### Baseline characteristics

This study included 62 consecutive eyes of 47 subjects that had a minimum postoperative follow-up duration of 8 years. The average age was 68.6 ± 8.8 years, and all eyes had a preoperative diagnosis of POAG, with mild severity in 64%, moderate in 16%, and severe in 20%. At baseline, the average IOP was 19.2 ± 3.9 mmHg, and the average medication use was 2.8 ± 1.1 glaucoma medications. The type and severity of cataract included nuclear sclerosis (NS) grade 1 (42%), NS grade 2 (36%), NS grade 3 (10%), cortical cataract (6%) and posterior subcapsular cataract (6%). Average BCVA was 0.17 ± 0.15 logMAR, and the average VF-MD was − 5.9 ± 6.6 dB. A total of 28 eyes (45.2%) had previously undergone SLT with a median time of 3.5 years (interquartile range [1.4–6.8] years) before CE-TMS. No eye had prior history of incisional glaucoma surgery. Demographic and baseline clinical patient characteristics are outlined in Table 1.

**Table 1:** Demographic and preoperative ocular characteristics

Variable	62 eyes of 47 subjects
Age at time of surgery (years)	68.6 ± 8.8
Sex (male:female), n (%)	29:33 (46.8:53.2)
Eye (OD:OS); n (%)	33:29 (53.2:46.8)
History of selective laser trabeculoplasty, n (%)	28 (45.2)
Central corneal thickness (μm)	545.7 ± 34.5
Axial length (mm)	24.0 ± 1.6
Anterior chamber depth (mm)	3.1 ± 0.4
Intraocular pressure (mmHg)	19.2 ± 3.9
Glaucoma medications	2.8 ± 1.1
Best-corrected visual acuity (logMAR)	0.17 ± 0.15
Cup-to-disc ratio	0.71 ± 0.16
Visual field mean deviation (dB)	− 5.9 ± 6.6
Retinal nerve fiber layer thickness (μm)	70.5 ± 13.7
Ganglion cell-inner plexiform layer thickness (μm)	64.4 ± 12.9

Mean ± standard deviations are presented, where applicable

### Efficacy measures

**Fig. 1:**
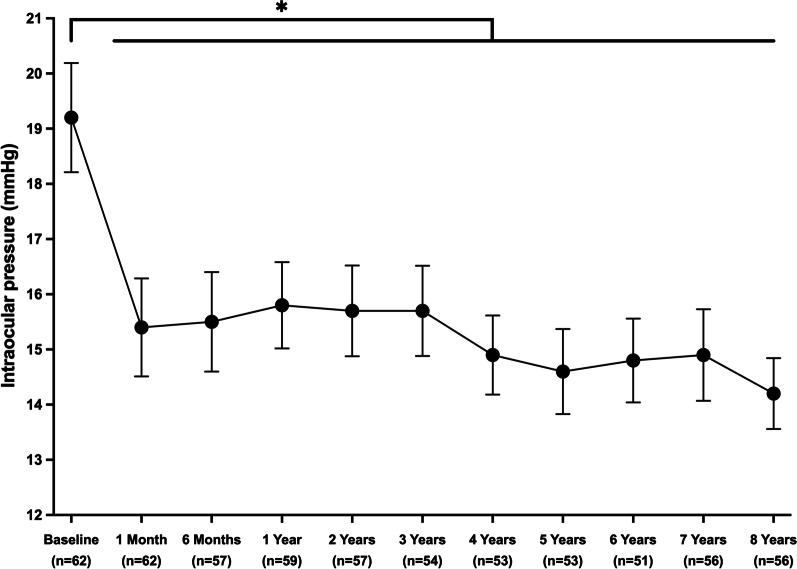
Change in intraocular pressure throughout eight years postoperative. * Denotes statistical significance at *P* < 0.05 and the error bars represent 95% confidence intervals. Mean ± standard deviations are presented

**Fig. 2:**
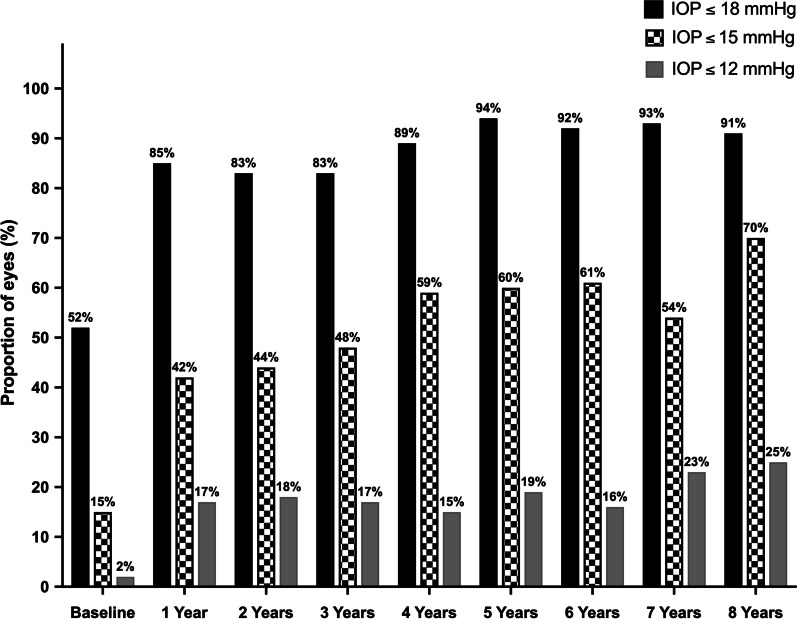
Eyes with IOP ≤ 18 mmHg, ≤ 15 mmHg, and ≤ 12 mmHg, preoperatively and throughout eight years postoperative. Bars represent the proportion of eyes with IOP ≤ 18 mmHg (solid black bars), IOP ≤ 15 mmHg (checked bars), and IOP ≤ 12 mmHg (solid grey bars). IOP: intraocular pressure

Efficacy measures consisted of postoperative reduction in IOP and glaucoma medication use, as well as surgical success. Postoperatively, a statistically significant reduction was observed in both IOP and glaucoma medication use, which persisted throughout the eight postoperative years (*P* < 0.001; Table 2). As illustrated in Fig. 1, IOP decreased significantly from 19.2 ± 3.9 mmHg preoperatively to 15.8 ± 3.0 mmHg at postoperative year (POY) 1 (3.4 mmHg absolute reduction, 17.7% relative reduction; *P* < 0.001), to 14.6 ± 2.8 mmHg at POY5 (4.6 mmHg absolute reduction, 24% relative reduction; *P* < 0.001), and to 14.2 ± 2.4 mmHg at POY8 (5.0 mmHg absolute reduction, 26% relative reduction; *P* < 0.001). Given the known association between the IOP-lowering effect of iStent and the baseline IOP, similar analyses were performed with baseline IOP as a covariate. While higher baseline IOP was significantly associated with a greater postoperative IOP reduction (*P* < 0.001), IOP change remained statistically significant at all postoperative time points (*P* < 0.001). Proportional analyses assessed the percentage of eyes with different IOPs (Fig. 2). Preoperatively, approximately half of the eyes (51.6%) had an IOP ≤ 18 mmHg, contrasting with 91.1% of eyes at POY8. In addition, only 14.5% of eyes had a preoperative IOP ≤ 15 mmHg, compared to more than two-thirds of eyes (69.6%) at POY8. Finally, IOP ≤ 12 mmHg was only noted in one eye (1.6%) preoperatively, whereas a quarter of eyes (25%) obtained this substantially low IOP at POY8.

**Table 2:** Eight-year outcomes in intraocular pressure, glaucoma medication use, and best-corrected visual acuity

Variable	N	Mean	% Change vs. baseline	Mean change vs. baseline	*P* value
**IOP (mmHg)**					
Preoperative	62	19.2 ± 3.9			
POM1	62	15.4 ± 3.5	− 19.8	− 3.8	< 0.001**
POM6	57	15.5 ± 3.4	− 19.3	− 3.7	< 0.001**
POY1	59	15.8 ± 3.0	− 17.7	− 3.4	< 0.001**
POY2	57	15.7 ± 3.1	− 18.2	− 3.5	< 0.001**
POY3	54	15.7 ± 3.0	− 18.2	− 3.5	< 0.001**
POY4	53	14.9 ± 2.6	− 22.4	− 4.3	< 0.001**
POY5	53	14.6 ± 2.8	− 24.0	− 4.6	< 0.001**
POY6	51	14.8 ± 2.7	− 22.9	− 4.4	< 0.001**
POY7	56	14.9 ± 3.1	− 22.4	− 4.3	< 0.001**
POY8	56	14.2 ± 2.4	− 26.0	− 5.0	< 0.001**
**Number of glaucoma medications**					
Preoperative	62	2.8 ± 1.1			
POM1	62	0.9 ± 1.3	− 67.9	− 1.9	< 0.001**
POM6	58	1.2 ± 1.2	− 57.1	− 1.5	< 0.001**
POY1	58	1.3 ± 1.2	− 53.6	− 1.5	< 0.001**
POY2	57	1.5 ± 1.2	− 46.4	− 1.3	< 0.001**
POY3	55	1.6 ± 1.3	− 42.9	− 1.1	< 0.001**
POY4	55	1.9 ± 1.3	− 32.1	− 0.9	< 0.001**
POY5	55	2.1 ± 1.2	− 25.0	− 0.8	< 0.001**
POY6	56	2.2 ± 1.2	− 21.4	− 0.6	< 0.001**
POY7	56	2.3 ± 1.2	− 17.9	− 0.6	0.002*
POY8	56	2.3 ± 1.2	− 17.9	− 0.5	0.006*
**BCVA (logMAR)**					
Preoperative	62	0.17 ± 0.15			
POM1	62	0.11 ± 0.10	− 35.3	− 0.06	< 0.001**
POM6	57	0.10 ± 0.19	− 41.2	− 0.07	< 0.001**
POY1	56	0.09 ± 0.11	− 47.1	− 0.08	< 0.001**
POY2	58	0.07 ± 0.10	− 58.8	− 0.10	< 0.001**
POY3	53	0.07 ± 0.10	− 58.8	− 0.10	< 0.001**
POY4	52	0.08 ± 0.11	− 52.9	− 0.09	< 0.001**
POY5	53	0.06 ± 0.09	− 64.7	− 0.11	< 0.001**
POY6	51	0.09 ± 0.11	− 47.1	− 0.08	< 0.001**
POY7	55	0.12 ± 0.12	− 29.4	− 0.05	0.014*
POY8	56	0.11 ± 0.14	− 35.3	− 0.06	0.015*

Mean ± standard deviations are presented and statistically compared to preoperative values using Generalized Estimating Equations with sequential Bonferroni correction for multiple comparisons

*IOP* intraocular pressure; *BCVA* best-corrected visual acuity; *POM* postoperative month; *POY* postoperative year

Statistical significance is denoted by * for *P* < 0.05 and ** for *P* < 0.001

**Fig. 3:**
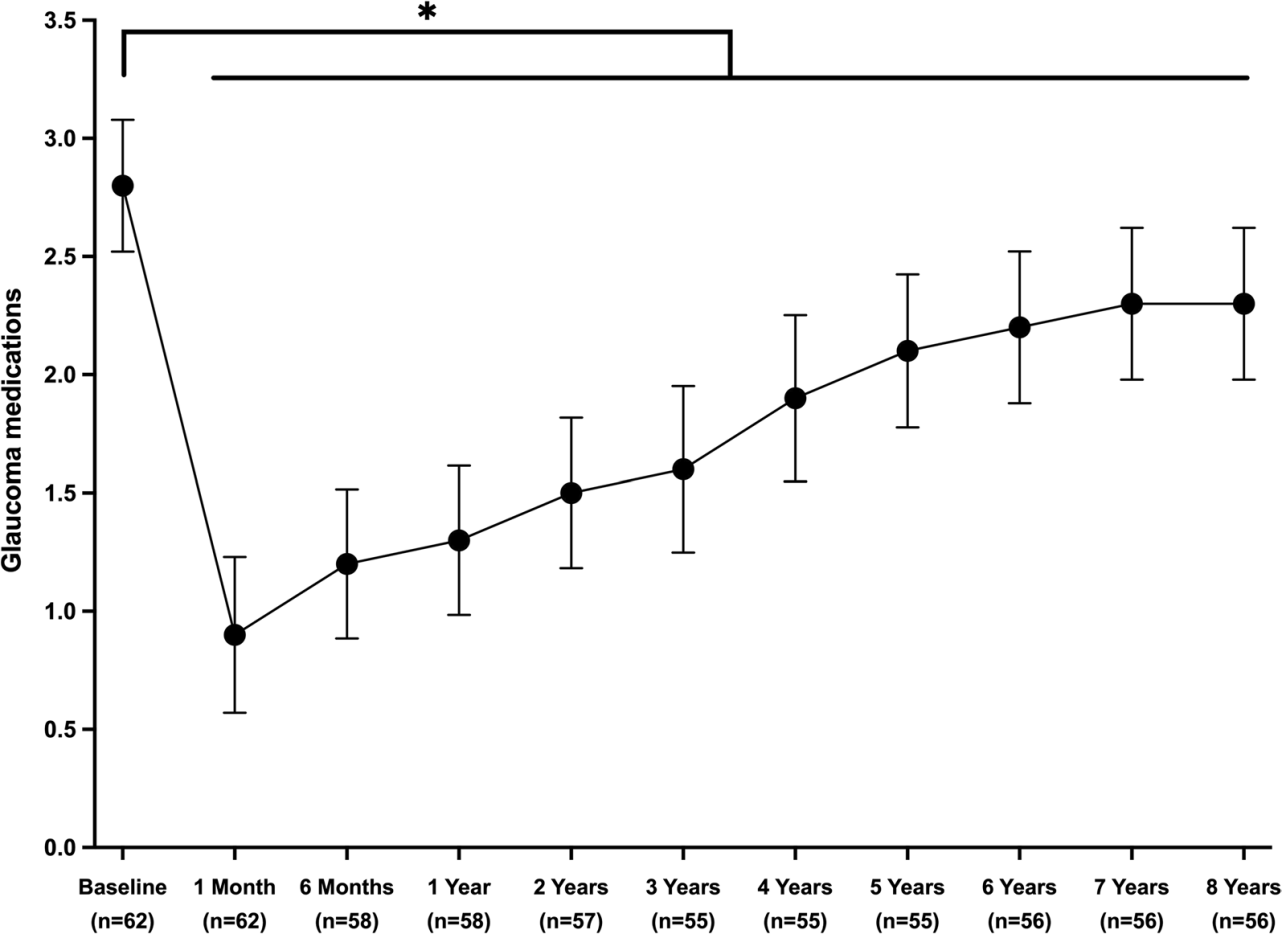
Change in the number of glaucoma medications throughout eight years postoperative. * Denotes statistical significance at *P* < 0.05 and the error bars represent 95% confidence intervals. Mean ± Standard deviations are presented

**Fig. 4:**
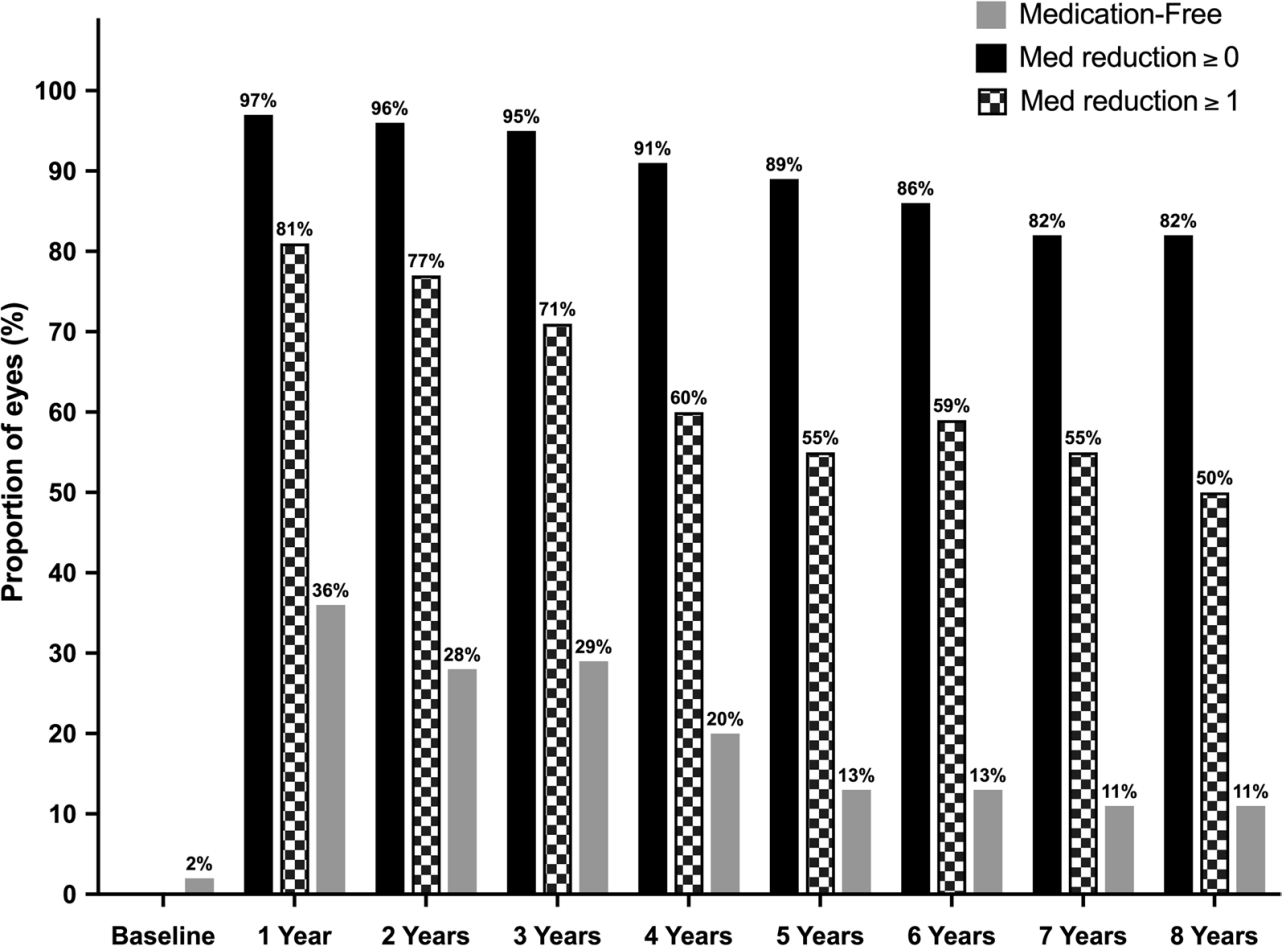
Medication-free eyes and eyes with reductions of ≥ 0 and ≥ 1 medication(s), preoperatively until eight years postoperative. Bars represent the proportion of medication-free eyes (solid grey bars), eyes with medication reduction of ≥ 0 medication(s) (solid black bars), and reduction of ≥ 1 medication(s) (checked bars)

As illustrated in Fig. 3, the number of glaucoma medications decreased from 2.8 ± 1.1 preoperatively to 0.9 ± 1.3 at postoperative month (POM) 1 (1.9 medications absolute reduction, 67.9% relative reduction; *P* < 0.001), then progressively increased to 1.3 ± 1.2 at POY1 (1.5 absolute reduction, 53.6% relative reduction; *P* < 0.001), 2.1 ± 1.2 at POY5 (0.8 absolute reduction, 25.0% relative reduction; *P* < 0.001), and finally, to 2.3 ± 1.2 at POY8 (0.5 absolute reduction, 17.9% relative reduction; *P* = 0.006). The proportion of medication-free eyes increased from 1.6% preoperatively to 36.2% at POY1, 12.7% at POY5, and 10.7% at POY8. At 8 years postoperative, the use of glaucoma medications was maintained or decreased in 82.1% of eyes, and half of the eyes had decreased their medication use by ≥ 1 medication(s) (Fig. 4).

**Fig. 5:**
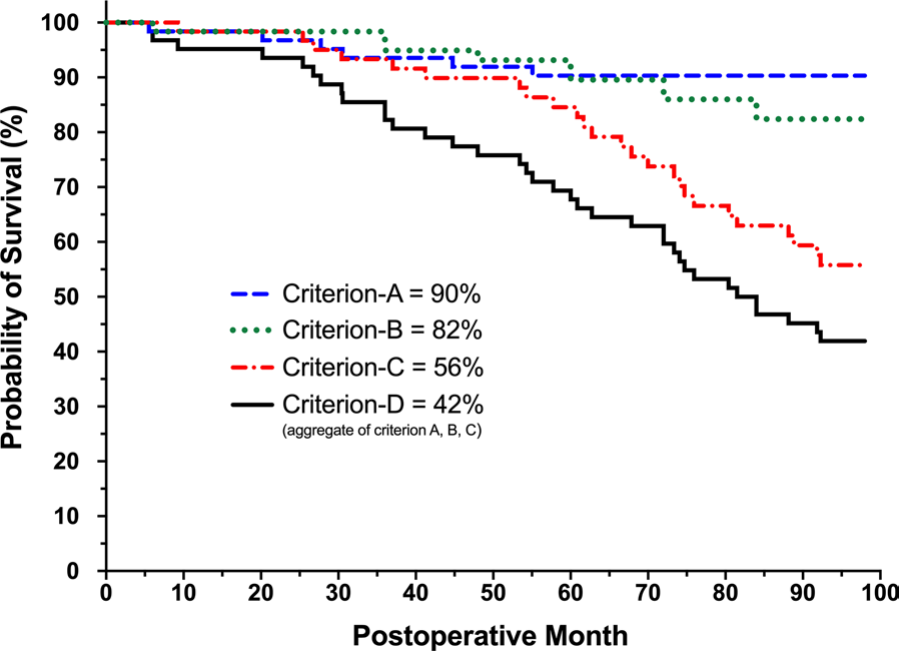
Eight-year Kaplan–Meier survival chart. The cumulative eight-year survival was 90% for Criterion-A (dashed blue line; glaucoma reoperation due to inadequate IOP control or disease progression), 82% for Criterion-B (dotted green line; increased use of glaucoma medications at ≥ 2 visits), 56% for Criterion-C (dashed and dotted red line; selective laser trabeculoplasty), and 42% for Criterion-D (aggregate of criterion A, B, and C; solid black line)

Kaplan–Meier survival curve illustrates the cumulative success according to four criteria (Fig. 5). According to Criterion-A, surgical success was 90%, as six eyes underwent subsequent glaucoma surgeries, including one with the Ahmed Glaucoma Valve (POM5), one with cyclophotocoagulation (POM20), three with non-penetrating glaucoma surgery (POM27, POM30, POM55), and one with the Hydrus microstent (POM44). According to Criterion-B, the 8-year surgical success was 82%, with 10 eyes failing due to an increase in the use of glaucoma medications at two or more consecutive visits compared to baseline. Criterion-C yielded a surgical success of 56%, with 25 eyes failing due to the need for SLT. Finally, Criterion-D (the aggregate of criteria A, B, and C)—yielded an 8-year surgical success of 42%.

### Safety measures

Postoperatively, BCVA experienced a statistically significant improvement which persisted throughout the eight years of follow-up (Table 2; *P* < 0.05). At POY8, all eyes had a BCVA of 20/100 or better, and 68.5% had a BCVA of 20/25 or better, compared to only 45.2% preoperatively. A small proportion of eyes (14.5%) experienced worsening of BCVA compared with their preoperative vision. The causes of vision decline in these nine eyes were attributed to the progression of age-related macular degeneration (five eyes), epiretinal membrane (two eyes), vitelliform macular dystrophy (one eye), and progression of glaucoma (one eye). Structural measures of disease stability remained stable throughout the 8-year follow-up (Table 3), including CDR (*P* = 0.103), RNFL thickness (*P* = 0.951), and GC-IPL thickness (*P* = 0.171). VF-MD remained stable up until POY5. However, compared to the preoperative VF-MD (− 5.9 ± 6.6 dB), this measure progressed at subsequent years, including POY6 (− 7.0 ± 7.1 dB; *P* = 0.027), POY7 (− 6.6 ± 7.3 dB; *P* = 0.043), and POY8 (− 8.0 ± 7.8 dB; *P* < 0.001).

**Table 3:** Eight-year outcomes in visual field mean deviation, cup-to-disc ratio, retinal nerve fiber layer thickness, and ganglion cell-inner plexiform layer thickness

Variable	N	Mean	% Change vs. baseline	Mean change vs. baseline	*P* value
**VF-MD (dB)**					
Preoperative	58	− 5.9 ± 6.6			
POY1	52	− 4.1 ± 5.8	− 30.5	1.8	0.092
POY2	46	− 4.7 ± 6.0	− 20.3	1.2	0.130
POY3	47	− 6.3 ± 6.5	6.8	− 0.4	0.227
POY4	46	− 5.8 ± 6.3	− 1.7	0.1	0.953
POY5	45	− 5.5 ± 6.4	− 6.8	0.4	0.634
POY6	44	− 7.0 ± 7.1	18.6	− 1.1	0.027*
POY7	45	− 6.6 ± 7.3	11.9	− 0.7	0.043*
POY8	57	− 8.0 ± 7.8	35.6	− 2.1	< 0.001**
**CDR**					
Preoperative	42	0.71 ± 0.16			
POY1	45	0.71 ± 0.16	0.0	0.00	0.751
POY2	52	0.70 ± 0.17	− 1.4	− 0.01	0.203
POY3	49	0.70 ± 0.17	− 1.4	− 0.01	0.322
POY4	48	0.70 ± 0.16	− 1.4	− 0.01	0.310
POY5	48	0.70 ± 0.16	− 1.4	− 0.01	0.187
POY6	47	0.71 ± 0.16	0.0	0.00	0.830
POY7	45	0.71 ± 0.15	0.0	0.00	0.782
POY8	53	0.70 ± 0.16	− 1.4	− 0.01	0.176
**RNFL thickness (μm)**					
Preoperative	14	70.5 ± 13.7			
POY1	19	71.7 ± 11.3	1.7	1.2	0.613
POY2	35	74.4 ± 13.5	5.5	3.9	0.205
POY3	36	74.7 ± 12.6	6.0	4.2	0.214
POY4	39	74.8 ± 11.3	6.1	4.3	0.192
POY5	42	74.7 ± 12.0	6.0	4.2	0.222
POY6	41	74.6 ± 12.6	5.8	4.1	0.230
POY7	40	75.2 ± 12.2	6.7	4.7	0.181
POY8	49	74.3 ± 11.9	5.4	3.8	0.228
**GC-IPL thickness (μm)**					
Preoperative	14	64.4 ± 12.9			
POY1	19	65.7 ± 11.2	2.0	1.3	0.623
POY2	34	65.6 ± 13.1	1.9	1.2	0.674
POY3	36	64.2 ± 11.9	− 0.3	− 0.2	0.962
POY4	39	67.4 ± 11.2	4.7	3.0	0.337
POY5	42	65.5 ± 11.5	1.7	1.1	0.679
POY6	41	64.2 ± 12.5	− 0.3	− 0.2	0.966
POY7	40	65.1 ± 11.4	1.1	0.7	0.752
POY8	48	63.8 ± 10.9	− 0.9	− 0.6	0.857

*VF-MD* visual field mean deviation; *CDR* cup-to-disc ratio; *RNFL* retinal nerve fiber layer; *GC-IPL* ganglion cell-inner plexiform layer; *POM* postoperative month; *POY* postoperative year

Mean ± Standard deviations are presented and statistically compared to preoperative values using Generalized Estimating Equations with sequential Bonferroni correction for multiple comparisons. Statistical significance is denoted by * for *P* < 0.05 and ** for *P* < 0.001

No intraoperative complications were noted in any of the eyes. With regards to postoperative adverse events, 18 eyes (29%) experienced IOP spikes, the majority of which (94%) occurred by postoperative week (POW) 2. Six eyes underwent an anterior chamber tap, and the spike in IOP was thought to be secondary to steroid response which improved upon tapering of the topical steroids in five eyes. Five eyes (8.1%) experienced transient micro-hyphema at postoperative day (POD) 1. One eye experienced transitory central macular edema which was managed by topical non-steroidal anti-inflammatory drugs. Posterior capsular opacification was noted in 27 eyes, nine of which underwent YAG capsulotomy during the 8-year follow-up. Of note, no eye experienced any sight-threatening complications such as endophthalmitis, hypotony, choroidal detachment, rebound iritis or stent obstruction, or loss of light perception throughout the 8-year follow-up.

### Supplementary analyses to address the missing data

The MCAR test confirmed that the missing data were missing completely at random (*P* = 0.543). Thus, multiple imputations were performed. Similar statistical analyses as reported above were performed using the imputed data. The results were comparable to those from the original dataset with missing data and are reported in Additional files 1, 2: Tables S1 and S2.

## Discussion

Over the past decade, the surgical management of glaucoma has been revolutionized. The ever-increasing wealth of data on the variety of MIGS procedures, most of which supports their efficacy and safety, has contributed to a paradigm shift in the surgical management of glaucoma. Given the novelty of MIGS, studies have often been limited to short- [59–62] or medium-term outcomes [28, 63–65]. Thus far, the longest-term study on iStent—the first FDA-approved MIGS device—reports outcomes up to 7 years postoperative [52]. Thus, the present, reasonably-sized 8-year outcome study contributes to the long-term literature on iStent. The results from a homogenous group of POAG eyes without prior incisional glaucoma surgeries support the long-term efficacy of iStent, while also demonstrating the safety of this procedure through a variety of markers for disease stability, some of which were omitted by similar long-term studies.

CE-TMS leads to a significant IOP reduction immediately postoperative, which was sustained throughout the 8 years of follow-up. At POY8, the cohort experienced an absolute IOP reduction of 5.0 mmHg—translating to a 26% relative reduction compared to the preoperative IOP (*P* < 0.001). Our 8-year results are notable as the long-term efficacy of iStent, and MIGS in general, remains an outstanding question [36]. Histopathological studies have evidenced inflammatory and fibrotic changes in the trabecular meshwork surrounding the trabecular micro-bypass stent [66]. Although it can be postulated that this inflammation and fibrosis due to the insertion of the iStent could lead to increased IOP, another study including 24 eyes showed no statistical difference in the 48-month IOP of eyes with combined iStent implantation and cataract surgery (n = 10) compared to those with cataract surgery alone (n = 14) [60]. The absence of intergroup differences could be due to the study’s small sample size, rendering the statistical analysis underpowered. Nevertheless, our data is supported by 19.9% and 22.9% reductions reported by prior studies at POY6 and POY7, respectively [51, 52]. The larger reduction evidenced in our cohort, which was also sustained over a longer follow-up period, could be attributed to multiple factors, including the inter-population differences and the use of an additional iStent (for a total of two stents) compared to the aforementioned studies. The use of a single stent has been shown to lead to significant IOP reductions; however, implantation of additional stents can lead to incrementally greater IOP reductions sustained over a longer period [67]. Another explanation could be the differences in surgical techniques. As detailed in our previous studies of the iStent inject, [15–18] the stents were implanted into the trabecular meshwork, adjacent to the collector channels (identified and marked preoperatively, as described in “Methods”), to ensure maximal proximity of the stents to the collector channel ostia. Our rationale is based on the knowledge that collector channel ostia provide the shortest pathway to aqueous veins and that the collector channels in these areas are more dilated, which collectively can allow for the most efficient outflow [68–70].

In addition to IOP reduction, the use of glaucoma medications decreased significantly as well. This decrease peaked at POM1, with an absolute reduction of 1.9 medications (67.9% relative reduction; *P* < 0.001). Subsequently, glaucoma medications were gradually reintroduced until POY8 with a final absolute reduction of 0.5 medication (17.9% relative reduction), which remained significantly less than the preoperative use. This is in keeping with the findings of the 6-year and 7-year studies, where they reported an average decrease of 0.4 and 0.6 medication, respectively [51, 52]. Of note, the use of glaucoma medications decreased by at least one medication in half of the eyes, and 10.7% were medication-free at POY8. This is particularly important in the context of known shortcomings of topical glaucoma drops including disruption of the ocular surface, limited potential in controlling the diurnal IOP variations, dependence on patient’s compliance, and cost—most of which are addressed by surgical interventions [4–6]. Of note, iStent implantation has been shown to improve quality of life in the context of decreased number of glaucoma medications [71].

The World Glaucoma Association consensus, detailed in Guidelines on Design and Reporting of Glaucoma, recommends studies to report surgical success through Kaplan–Meier graphs [72]. With regards to MIGS, success is typically defined as postoperative IOP below a certain cut-off and a minimum IOP reduction of 20%. In the absence of preoperative medication washout, we believe that inclusion of such criteria for a cohort of medically controlled patients with a preoperative IOP of 19.2 ± 3.9 mmHg, where a minimum IOP reduction of 20% translates to an IOP range close to the episcleral venous pressure, would lead to mislabeling of a majority of the eyes as failures. Thus, we defined four failure criteria as described in the Methods section. Our Kaplan–Meier survival analyses demonstrated an excellent 8-year survival of 90% according to Criterion-A, as only six eyes underwent additional glaucoma surgeries throughout the follow-up. Also, the number of glaucoma medications increased in 10 eyes and 25 eyes underwent SLT, resulting in success rates of 82% and 56% for Criterion-B and Criterion-C, respectively. It is important to interpret the postoperative IOP drop in the context of additional interventions such as SLT or glaucoma drops for those with suboptimal postoperative response. The 10% reoperation rate in our study is in line with that of Ziaei et al. where a total of 5 eyes (12%) underwent further glaucoma surgeries throughout the 7-year follow-up [52]. This low reoperation rate along with the sustained IOP reduction throughout the 8 years of follow-up supports the long-term efficacy of iStent, contrasting to the shorter-term decrease in IOP achieved with SLT or cataract surgery [73–76].

CE-TMS showed excelled safety throughout 8 years postoperative. Early postoperative IOP spike was the most common adverse event. A recent study by Salimi et al., found that limiting the use of postoperative steroids in CE-TMS can help minimize early postoperative IOP spikes [34]. No evidence of stent obstruction or stent-associated complication was noted and none of the cases experienced any sight-threatening adverse event. The postoperative improvements in BCVA persisted throughout the follow-up, and all eyes had a vision equal to or better than 20/100. While this is attributable to the vision-enhancing effect of cataract surgery, the absence of cases with vision worse than 20/100 or loss of light perception supports the safety of CE-TMS. VF-MD—a functional measure of disease progression—remained stable until POY5 and progressed thereafter. In contrast to our results, Ziaei et al., did not find any significant differences between the VF-MD at baseline and POY7 [52]. However, in their study, only 19 patients had 7-year outcomes, which could explain the lack of significant findings due to a potentially underpowered analysis. Glaucoma is a progressive disease and in the absence of curative treatment, all currently available interventions aim to slow down the rate of disease progression. Thus, our findings with regards to VF-MD, along with those of Ziaei et al., are valuable findings supporting the long-term efficacy of iStent in stabilizing or slowing disease progression. All the structural markers of disease progression remained stable postoperatively, including CDR, RNFL thickness, and GC-IPL thickness. Previous studies have evidenced a poor agreement between progression detection of RNFL, neuroretinal rim, and visual field measurements [77]. In this context, we encourage future studies to systematically report VF-MD, RNFL, and GC-IPL data—measures that are not consistently reported by MIGS studies.

Our study is not without limitations. The retrospective nature of the study, while provides valuable real-world long-term data, had the potential for containing missing data. To address this shortcoming, after confirming that the data were missing completely at random, we performed secondary analyses on an imputed database that evidenced very similar findings (Additional files 1, 2: Tables S1 and S2). The absence of a control arm limits our findings to the efficacy of a combined procedure, as opposed to iStent as a stand-alone procedure. Medication washout was not performed for any of the eyes, as this can be inappropriate in a real-world clinical setting. It can be argued that the absence of preoperative medication washout is a strength, as it makes the degree of IOP and medication reduction more noteworthy. Our cohort consisted of medically controlled glaucomatous eyes with no prior incisional glaucoma surgery; thus, our findings may not necessarily be generalizable to eyes with advanced, uncontrolled glaucoma—a subpopulation that should be studied by future works. In absence of available information regarding the ethnic background of patients, we were unable to assess potential associations between ethnicity and surgical outcomes. Finally, given the retrospective and unmasked nature of this study, we cannot rule out the possibility of investigator bias in performing postoperative measurements. While this is a limitation, the single-surgeon nature of the study limits inter-rater variability. Identification of the risk factors for early postoperative failure that requires surgical reoperation will be of interest to future research.

## Conclusion

This 8-year study provides the longest follow-up data, available to date, on the efficacy and safety of cataract surgery with concurrent implantation of two iStents, using a reasonably sized and homogenous cohort of POAG eyes. The results support the long-term efficacy of CE-TMS in reducing IOP and glaucoma medication use, while also improving visual acuity throughout 8 years postoperative. Furthermore, functional and structural markers of disease stability supported the long-term efficacy of this procedure in stabilizing or slowing disease progression. These findings, along with the excellent safety profile evidenced, suggest that the insertion of two iStents combined with cataract surgery is an efficacious and safe option for surgery naïve POAG eyes, with results lasting up to 8 years postoperative.

## Supplementary Information

**Additional file 1: Table S1.** Eight-year outcomes in intraocular pressure, glaucoma medication use, and best-corrected visual acuity, using imputed data.

**Additional file 2: Table S2. **Eight-year outcomes in visual field mean deviation, cup-to-disc ratio, retinal nerve fiber layer thickness, and ganglion cell-inner plexiform layer thickness, using imputed data.

## Data Availability

The data that support the findings of this study are available from the corresponding author upon reasonable request.

## Abbreviations

BCVA: Best-corrected visual acuity
CE-TMS: Cataract extraction with implantation of trabecular micro-bypass stents
CDR: Cup-to-disc ratio
FDA: Food and Drug Association
GC-IPL: Ganglion cell-inner plexiform layer
GEE: Generalized estimating equations
IOP: Intraocular pressure
LogMAR: Logarithm of the minimum angle of resolution
MIGS: Minimally invasive glaucoma surgeries
MCAR: Missing completely at random
POAG: Primary open-angle glaucoma
RNFL: Retinal nerve fiber layer
VF-MD: Visual field mean deviation

## References

[1] Weinreb RN, Aung T, Medeiros FA (2014). The pathophysiology and treatment of glaucoma: a review. JAMA.

[2] Tham YC, Li X, Wong TY, Quigley HA, Aung T, Cheng CY (2014). Global prevalence of glaucoma and projections of glaucoma burden through 2040: a systematic review and meta-analysis. Ophthalmology.

[3] Harasymowycz P, Birt C, Gooi P, Heckler L, Hutnik C, Jinapriya D (2016). Medical management of glaucoma in the 21st century from a Canadian perspective. J Ophthalmol.

[4] Baudouin C, Labbe A, Liang H, Pauly A, Brignole-Baudouin F (2010). Preservatives in eyedrops: the good, the bad and the ugly. Prog Retin Eye Res.

[5] Leung EW, Medeiros FA, Weinreb RN (2008). Prevalence of ocular surface disease in glaucoma patients. J Glaucoma.

[6] Joseph A, Pasquale LR (2017). Attributes associated with adherence to glaucoma medical therapy and its effects on glaucoma outcomes: an evidence-based review and potential strategies to improve adherence. Semin Ophthalmol.

[7] Jampel HD, Musch DC, Gillespie BW, Lichter PR, Wright MM, Guire KE (2005). Perioperative complications of trabeculectomy in the collaborative initial glaucoma treatment study (CIGTS). Am J Ophthalmol.

[8] Gedde SJ, Herndon LW, Brandt JD, Budenz DL, Feuer WJ, Schiffman JC (2012). Postoperative complications in the Tube Versus Trabeculectomy (TVT) study during five years of follow-up. Am J Ophthalmol.

[9] Rulli E, Biagioli E, Riva I, Gambirasio G, De Simone I, Floriani I (2013). Efficacy and safety of trabeculectomy vs nonpenetrating surgical procedures: a systematic review and meta-analysis. JAMA Ophthalmol.

[10] Rosdahl JA, Gupta D (2020). Prospective studies of minimally invasive glaucoma surgeries: systematic review and quality assessment. Clin Ophthalmol.

[11] Shah M (2019). Micro-invasive glaucoma surgery—an interventional glaucoma revolution. Eye Vis (Lond).

[12] Saheb H, Ahmed II (2012). Micro-invasive glaucoma surgery: current perspectives and future directions. Curr Opin Ophthalmol.

[13] Samuelson TW, Katz LJ, Wells JM, Duh YJ, Giamporcaro JE, US iStent Study Group (2011). Randomized evaluation of the trabecular micro-bypass stent with phacoemulsification in patients with glaucoma and cataract. Ophthalmology.

[14] Fernandez-Barrientos Y, Garcia-Feijoo J, Martinez-de-la-Casa JM, Pablo LE, Fernandez-Perez C, García Sánchez J (2010). Fluorophotometric study of the effect of the glaukos trabecular microbypass stent on aqueous humor dynamics. Invest Ophthalmol Vis Sci.

[15] Salimi A, Abu-Nada M, Harasymowycz P (2021). Matched cohort study of cataract surgery with and without trabecular microbypass stent implantation in primary angle-closure glaucoma. Am J Ophthalmol.

[16] Salimi A, Watt H, Harasymowycz P (2021). Three-year outcomes of second-generation trabecular micro-bypass stents (iStent inject) with phacoemulsification in various glaucoma subtypes and severities. J Glaucoma.

[17] Salimi A, Clement C, Shiu M, Harasymowycz P (2020). Second-generation trabecular micro-bypass (iStent inject) with cataract surgery in eyes with normal-tension glaucoma: one-year outcomes of a multi-centre study. Ophthalmol Ther.

[18] Salimi A, Lapointe J, Harasymowycz P (2019). One-year outcomes of second-generation trabecular micro-bypass stents (iStent Inject) implantation with cataract surgery in different glaucoma subtypes and severities. Ophthalmol Ther.

[19] Resende AF, Patel NS, Waisbourd M, Katz LJ (2016). iStent(R) trabecular microbypass stent: an update. J Ophthalmol.

[20] El Wardani M, Bergin C, Achache F, Sharkawi E (2015). Evaluating the trabecular micro-bypass stent combined with phacoemulsification compared to phacoemulsification alone. Klin Monbl Augenheilkd.

[21] Ahmed II, Katz LJ, Chang DF, Donnenfeld ED, Solomon KD, Voskanyan L (2014). Prospective evaluation of microinvasive glaucoma surgery with trabecular microbypass stents and prostaglandin in open-angle glaucoma. J Cataract Refract Surg.

[22] Fea AM, Belda JI, Rekas M, Junemann A, Chang L, Pablo L (2014). Prospective unmasked randomized evaluation of the iStent inject (R) versus two ocular hypotensive agents in patients with primary open-angle glaucoma. Clin Ophthalmol.

[23] Voskanyan L, Garcia-Feijoo J, Belda JI, Fea A, Junemann A, Baudouin C (2014). Prospective, unmasked evaluation of the iStent® inject system for open-angle glaucoma: synergy trial. Adv Ther.

[24] Arriola-Villalobos P, Martinez-de-la-Casa JM, Diaz-Valle D, Garcia-Vidal SE, Fernandez-Perez C, Garcia-Sanchez J (2013). Mid-term evaluation of the new Glaukos iStent with phacoemulsification in coexistent open-angle glaucoma or ocular hypertension and cataract. Br J Ophthalmol.

[25] Patel I, de Klerk TA, Au L (2013). Manchester iStent study: early results from a prospective UK case series. Clin Exp Ophthalmol.

[26] Belovay GW, Naqi A, Chan BJ, Rateb M, Ahmed II (2012). Using multiple trabecular micro-bypass stents in cataract patients to treat open-angle glaucoma. J Cataract Refract Surg.

[27] Craven ER, Katz LJ, Wells JM, Giamporcaro JE, iStent Study Group (2012). Cataract surgery with trabecular micro-bypass stent implantation in patients with mild-to-moderate open-angle glaucoma and cataract: two-year follow-up. J Cataract Refract Surg.

[28] Arriola-Villalobos P, Martinez-de-la-Casa JM, Diaz-Valle D, Fernandez-Perez C, Garcia-Sanchez J, Garcia-Feijoo J (2012). Combined iStent trabecular micro-bypass stent implantation and phacoemulsification for coexistent open-angle glaucoma and cataract: a long-term study. Br J Ophthalmol.

[29] Buchacra O, Duch S, Milla E, Stirbu O (2011). One-year analysis of the iStent trabecular microbypass in secondary glaucoma. Clin Ophthalmol.

[30] Fea AM (2010). Phacoemulsification versus phacoemulsification with micro-bypass stent implantation in primary open-angle glaucoma: randomized double-masked clinical trial. J Cataract Refract Surg.

[31] Spiegel D, Wetzel W, Neuhann T, Stuermer J, Hoeh H, Garcia-Feijoo J (2009). Coexistent primary open-angle glaucoma and cataract: interim analysis of a trabecular micro-bypass stent and concurrent cataract surgery. Eur J Ophthalmol.

[32] Spiegel D, Garcia-Feijoo J, Garcia-Sanchez J, Lamielle H (2008). Coexistent primary open-angle glaucoma and cataract: preliminary analysis of treatment by cataract surgery and the iStent trabecular micro-bypass stent. Adv Ther.

[33] Spiegel D, Wetzel W, Haffner DS, Hill RA (2007). Initial clinical experience with the trabecular micro-bypass stent in patients with glaucoma. Adv Ther.

[34] Salimi A, Winter A, Li C, Harasymowycz P, Saheb H (2019). Effect of topical corticosteroids on early postoperative intraocular pressure following combined cataract and trabecular microbypass surgery. J Ocul Pharmacol Ther.

[35] Nieland K, Labbe A, Schweitzer C, Gicquel G, Kleintjens J, Ostawal A (2021). A cost-effectiveness analysis of iStent inject combined with phacoemulsification cataract surgery in patients with mild-to-moderate open-angle glaucoma in France. PLoS One.

[36] Jablonska J, Lewczuk K, Konopinska J, Mariak Z, Rękas M (2021). Microinvasive glaucoma surgery: a review and classification of implant-dependent procedures and techniques. Acta Ophthalmol.

[37] Paletta Guedes RA, Gravina DM, Paletta Guedes VM, Chaoubah A (2021). Two-year comparative outcomes of first- and second-generation trabecular micro-bypass stents with cataract surgery. Clin Ophthalmol.

[38] Kozera M, Konopinska J, Rękas M (2021). Mid-term evaluation of the safety and efficacy of the iStent trabecular micro-bypass system combined with phacoemulsification. Adv Clin Exp Med.

[39] Seixas SRC, Balbino M, Basile Neto A, de Costa AAA, Jordao M, Russ HHA (2020). Mid-term evaluation of iStent inject® trabecular micro-bypass stent implantation with or without phacoemulsification: a retrospective study. Clin Ophthalmol.

[40] Nitta K, Yamada Y, Morokado S, Sugiyama K (2020). iStent trabecular micro-bypass stent implantation with cataract surgery in a Japanese glaucoma population. Clin Ophthalmol.

[41] Al Habash A, Khan O (2020). Outcomes of combined iStent trabecular micro-bypass and cataract surgery for the treatment of open-angle glaucoma in a Saudi population. Clin Ophthalmol.

[42] Ferguson TJ, Dockter Z, Bleeker A, Karpuk KL, Schweitzer J, Ibach MJ (2020). iStent inject trabecular microbypass stent implantation with cataract extraction in open-angle glaucoma: early clinical experience. Eye Vis (Lond).

[43] Berdahl J, Voskanyan L, Myers JS, Katz LJ, Samuelson TW (2020). iStent inject trabecular micro-bypass stents with topical prostaglandin as standalone treatment for open-angle glaucoma: 4-year outcomes. Clin Exp Ophthalmol.

[44] Lindstrom R, Sarkisian SR, Lewis R, Hovanesian J, Voskanyan L (2020). Four-year outcomes of two second-generation trabecular micro-bypass stents in patients with open-angle glaucoma on one medication. Clin Ophthalmol.

[45] Neuhann R, Neuhann T (2020). Second-generation trabecular micro-bypass stent implantation: retrospective analysis after 12- and 24-month follow-up. Eye Vis (Lond).

[46] Ferguson TJ, Ibach M, Schweitzer J, Karpuk KL, Stephens JD, Berdahl JP (2020). Trabecular micro-bypass stent implantation with cataract extraction in pigmentary glaucoma. Clin Exp Ophthalmol.

[47] Manning D (2019). Real-world case series of iStent or iStent inject trabecular micro-bypass stents combined with cataract surgery. Ophthalmol Ther.

[48] Samuelson TW, Sarkisian SR Jr, Lubeck DM, Stiles MC, Duh YJ, Romo EA, et al. Prospective, randomized, controlled pivotal trial of an ab interno implanted trabecular micro-bypass in primary open-angle glaucoma and cataract: two-year results. Ophthalmology. 2019;126(6):811–21. 10.1016/j.ophtha.2019.03.006 30880108

[49] Ferguson T, Swan R, Ibach M, Schweitzer J, Sudhagoni R, Berdahl JP (2018). Evaluation of a trabecular microbypass stent with cataract extraction in severe primary open-angle glaucoma. J Glaucoma.

[50] Neuhann TH (2015). Trabecular micro-bypass stent implantation during small-incision cataract surgery for open-angle glaucoma or ocular hypertension: long-term results. J Cataract Refract Surg.

[51] Ferguson TJ, Mechels KB, Dockter Z, Bleeker A, Ibach M, Schweitzer J (2020). iStent trabecular microbypass stent implantation with phacoemulsification in patients with open-angle glaucoma: 6-year outcomes. Clin Ophthalmol.

[52] Ziaei H, Au L. Manchester iStent study: long-term 7-year outcomes. Eye (Lond). 2020;35(8):2277–82. 10.1038/s41433-020-01255-6 PMC8302602 33139875

[53] Chylack LT Jr, Wolfe JK, Singer DM, Leske MC, Bullimore MA, Bailey IL, et al. The Lens Opacities Classification System III. The longitudinal study of cataract study group. Arch Ophthalmol. 1993;111(6):831–6. 10.1001/archopht.1993.01090060119035 8512486

[54] Hodapp E, Parrish RK, Anderson DR. Clinical decisions in glaucoma: Mosby Incorporated; 1993.

[55] Mwanza JC, Durbin MK, Budenz DL, Girkin CA, Leung CK, Liebmann JM (2011). Profile and predictors of normal ganglion cell-inner plexiform layer thickness measured with frequency-domain optical coherence tomography. Invest Ophthalmol Vis Sci.

[56] Salimi A, Nithianandan H, Al Farsi H, Harasymowycz P, Saheb H (2021). Gonioscopy-assisted transluminal trabeculotomy in younger to middle-aged adults: one-year outcomes. Ophthalmol Glaucoma.

[57] Salimi A, Kovalyuk N, Harasymowycz PJ (2019). Tube shunt revision with excision of fibrotic capsule using mitomycin c with and without ologen-a collagen matrix implant: a 3-year follow-up study. J Glaucoma.

[58] Wang M (2014). Generalized estimating equations in longitudinal data analysis: a review and recent developments. Adv Stat.

[59] Myers JS, Masood I, Hornbeak DM, Belda JI, Auffarth G, Junemann A (2018). Prospective evaluation of two iStent((R)) trabecular stents, one iStent supra((R)) suprachoroidal stent, and postoperative prostaglandin in refractory glaucoma: 4-year outcomes. Adv Ther.

[60] Fea AM, Consolandi G, Zola M, Pignata G, Cannizzo P, Lavia C (2015). Micro-bypass implantation for primary open-angle glaucoma combined with phacoemulsification: 4-year follow-up. J Ophthalmol.

[61] Donnenfeld ED, Solomon KD, Voskanyan L, Chang DF, Samuelson TW, Ahmed II (2015). A prospective 3-year follow-up trial of implantation of two trabecular microbypass stents in open-angle glaucoma. Clin Ophthalmol.

[62] Chang DF, Donnenfeld ED, Katz LJ, Voskanyan L, Ahmed II, Samuelson TW (2017). Efficacy of two trabecular micro-bypass stents combined with topical travoprost in open-angle glaucoma not controlled on two preoperative medications: 3-year follow-up. Clin Ophthalmol.

[63] Saheb H, Donnenfeld ED, Solomon KD, Voskanyan L, Chang DF, Samuelson TW (2021). Five-year outcomes prospective study of two first-generation trabecular micro-bypass stents (iStent(R)) in open-angle glaucoma. Curr Eye Res.

[64] Neuhann TH, Hornbeak DM, Neuhann RT, Giamporcaro JE (2019). Long-term effectiveness and safety of trabecular microbypass stent implantation with cataract surgery in patients with glaucoma or ocular hypertension: five-year outcomes. J Cataract Refract Surg.

[65] Fechtner RD, Voskanyan L, Vold SD, Tetz M, Auffarth G, Masood I (2019). Five-year, prospective, randomized, multi-surgeon trial of two trabecular bypass stents versus prostaglandin for newly diagnosed open-angle glaucoma. Ophthalmol Glaucoma.

[66] Capitena Young CE, Ammar DA, Seibold LK, Pantcheva MB, SooHoo JR, Kahook MY (2018). Histopathologic examination of trabecular meshwork changes after trabecular bypass stent implantation. J Glaucoma.

[67] Katz LJ, Erb C, Carceller Guillamet A, Fea AM, Voskanyan L, Giamporcaro JE (2018). Long-term titrated IOP control with one, two, or three trabecular micro-bypass stents in open-angle glaucoma subjects on topical hypotensive medication: 42-month outcomes. Clin Ophthalmol.

[68] Hann CR, Bentley MD, Vercnocke A, Ritman EL, Fautsch MP (2011). Imaging the aqueous humor outflow pathway in human eyes by three-dimensional micro-computed tomography (3D micro-CT). Exp Eye Res.

[69] Hann CR, Fautsch MP (2009). Preferential fluid flow in the human trabecular meshwork near collector channels. Invest Ophthalmol Vis Sci.

[70] Battista SA, Lu Z, Hofmann S, Freddo T, Overby DR, Gong H (2008). Reduction of the available area for aqueous humor outflow and increase in meshwork herniations into collector channels following acute IOP elevation in bovine eyes. Invest Ophthalmol Vis Sci.

[71] Al Habash A, Nagshbandi AA (2020). Quality of life after combined cataract and minimally invasive glaucoma surgery in glaucoma patients. Clin Ophthalmol.

[72] Heuer DK, Barton K, Grehn F, Shaarawy T, Sherwood M, Shaarway T, Sherwood M, Grehn F (2009). Consensus on definition of success. Guidelines on Design and Reporting of Glaucoma Surgical Trials: World Glaucoma Association.

[73] Gazzard G, Konstantakopoulou E, Garway-Heath D, Garg A, Vickerstaff V, Hunter R (2019). Selective laser trabeculoplasty versus eye drops for first-line treatment of ocular hypertension and glaucoma (LiGHT): a multicentre randomised controlled trial. Lancet.

[74] Vizzeri G, Weinreb RN (2010). Cataract surgery and glaucoma. Curr Opin Ophthalmol.

[75] Poley BJ, Lindstrom RL, Samuelson TW (2008). Long-term effects of phacoemulsification with intraocular lens implantation in normotensive and ocular hypertensive eyes. J Cataract Refract Surg.

[76] Shingleton BJ, Gamell LS, O'Donoghue MW, Baylus SL, King R (1999). Long-term changes in intraocular pressure after clear corneal phacoemulsification: normal patients versus glaucoma suspect and glaucoma patients. J Cataract Refract Surg.

[77] Leung CK, Liu S, Weinreb RN, Lai G, Ye C, Cheung CY (2011). Evaluation of retinal nerve fiber layer progression in glaucoma a prospective analysis with neuroretinal rim and visual field progression. Ophthalmology.

