# Supplementary material for: Long-term outcomes of two first-generation trabecular micro-bypass stents (iStent) with phacoemulsification in primary open-angle glaucoma: eight-year results
Source: Eye Vis (Lond). 2021 Nov 16;8:43. doi: 10.1186/s40662-021-00263-1 (PMC8594216; doi:10.1186/s40662-021-00263-1)
Supplement: Supplementary file 1 — Additional file 1: Table S1. Eight-year outcomes in intraocular pressure, glaucoma medication use, and best-corrected visual acuity, using imputed data. [file 40662_2021_263_MOESM1_ESM.docx]

**Table S1.** Eight-year outcomes in intraocular pressure, glaucoma medication use, and best-corrected visual acuity, using imputed data.

|  | **Variable** | **N** | **Mean** | **% Change v*s.* Baseline** | **Mean change**  ***vs.* Baseline** | ***P*-value** |
| --- | --- | --- | --- | --- | --- | --- |
| IOP  (mmHg) | Preoperative | 62 | 19.2 ± 3.9 |  |  |  |
|  | POM1 | 62 | 15.4 ± 3.5 | −19.8 | −3.8 | <0.001** |
|  | POM6 | 61 | 15.5 ± 3.3 | −19.3 | −3.7 | <0.001** |
|  | POY1 | 61 | 15.8 ± 3.0 | −17.7 | −3.4 | <0.001** |
|  | POY2 | 60 | 15.7 ± 3.0 | −18.2 | −3.5 | <0.001** |
|  | POY3 | 58 | 15.6 ± 2.9 | −18.8 | −3.6 | <0.001** |
|  | POY4 | 57 | 14.9 ± 2.5 | −22.4 | −4.3 | <0.001** |
|  | POY5 | 56 | 14.6 ± 2.7 | −24.0 | −4.6 | <0.001** |
|  | POY6 | 56 | 14.8 ± 2.6 | −22.9 | −4.4 | <0.001** |
|  | POY7 | 56 | 14.9 ± 3.0 | −22.4 | −4.3 | <0.001** |
|  | POY8 | 56 | 14.2 ± 2.4 | −26.0 | −5.0 | <0.001** |
| Number of  glaucoma medications | Preoperative | 62 | 2.8 ± 1.1 |  |  |  |
|  | POM1 | 62 | 0.9 ± 1.3 | −67.9 | −1.9 | <0.001** |
|  | POM6 | 61 | 1.2 ± 1.2 | −57.1 | −1.6 | <0.001** |
|  | POY1 | 61 | 1.3 ± 1.2 | −53.6 | −1.5 | <0.001** |
|  | POY2 | 60 | 1.5 ± 1.2 | −46.4 | −1.3 | <0.001** |
|  | POY3 | 58 | 1.6 ± 1.3 | −42.9 | −1.2 | <0.001** |
|  | POY4 | 57 | 1.9 ± 1.3 | −32.1 | −0.9 | <0.001** |
|  | POY5 | 56 | 2.0 ± 1.2 | −28.6 | −0.8 | <0.001** |
|  | POY6 | 56 | 2.2 ± 1.2 | −21.4 | −0.6 | 0.001* |
|  | POY7 | 56 | 2.3 ± 1.2 | −17.9 | −0.5 | 0.002* |
|  | POY8 | 56 | 2.3 ± 1.2 | −17.9 | −0.5 | 0.006* |
| BCVA (logMAR) | Preoperative | 62 | 0.17 ± 0.15 |  |  |  |
|  | POM1 | 62 | 0.11 ± 0.14 | −35.3 | −0.06 | <0.001** |
|  | POM6 | 61 | 0.10 ± 0.20 | −41.2 | −0.07 | 0.004* |
|  | POY1 | 61 | 0.13 ± 0.35 | −23.5 | −0.04 | 0.037* |
|  | POY2 | 60 | 0.06 ± 0.10 | −64.7 | −0.11 | <0.001** |
|  | POY3 | 58 | 0.08 ± 0.11 | −52.9 | −0.09 | <0.001** |
|  | POY4 | 57 | 0.09 ± 0.12 | −47.1 | −0.08 | 0.001* |
|  | POY5 | 56 | 0.06 ± 0.09 | −64.7 | −0.11 | <0.001** |
|  | POY6 | 56 | 0.10 ± 0.12 | −41.2 | −0.07 | 0.002* |
|  | POY7 | 56 | 0.12 ± 0.11 | −29.4 | −0.05 | 0.015* |
|  | POY8 | 56 | 0.11 ± 0.13 | −35.3 | −0.06 | 0.010* |

*IOP* intraocular pressure; *BCVA* best-corrected visual acuity; *POM* postoperative month; *POY* postoperative year

Mean ± standard deviations are presented and statistically compared to preoperative values using Generalized Estimating Equations with sequential Bonferroni correction for multiple comparisons

Statistical significance is denoted by * for *P*<0.05 and ** for *P*<0.001
