# Supplementary material for: Long-term outcomes of two first-generation trabecular micro-bypass stents (iStent) with phacoemulsification in primary open-angle glaucoma: eight-year results
Source: Eye Vis (Lond). 2021 Nov 16;8:43. doi: 10.1186/s40662-021-00263-1 (PMC8594216; doi:10.1186/s40662-021-00263-1)
Supplement: Supplementary file 2 — Additional file 2: Table S2. Eight-year outcomes in visual field mean deviation, cup-to-disc ratio, retinal nerve fiber layer thickness, and ganglion cell-inner plexiform layer thickness, using imputed data. [file 40662_2021_263_MOESM2_ESM.docx]

**Table S2.** Eight-year outcomes in visual field mean deviation, cup-to-disc ratio, retinal nerve fiber layer thickness, and ganglion cell-inner plexiform layer thickness, using imputed data.

|  | **Variable** | **N** | **Mean** | **% Change v*s.* Baseline** | **Mean change**  ***vs.* Baseline** | ***P*-value** |
| --- | --- | --- | --- | --- | --- | --- |
| VF-MD (dB) | Preoperative | 62 | −5.9 ± 6.3 |  |  |  |
|  | POY1 | 61 | −4.2 ± 5.4 | −28.8 | 1.7 | 0.001* |
|  | POY2 | 60 | −4.7 ± 5.4 | −20.3 | 1.2 | 0.063 |
|  | POY3 | 58 | −6.4 ± 5.9 | 8.5 | −0.5 | 0.258 |
|  | POY4 | 57 | −5.7 ± 5.7 | −3.4 | 0.2 | 0.798 |
|  | POY5 | 56 | −5.6 ± 5.8 | −5.1 | 0.3 | 0.657 |
|  | POY6 | 56 | −7.1 ± 6.4 | 20.3 | −1.2 | 0.038* |
|  | POY7 | 56 | −6.6 ± 6.6 | 11.9 | −0.7 | 0.048* |
|  | POY8 | 56 | −7.7 ± 7.4 | 30.5 | −1.8 | 0.007* |
| CDR | Preoperative | 62 | 0.69 ± 0.20 |  |  |  |
|  | POY1 | 61 | 0.70 ± 0.19 | 1.4 | 0.01 | 0.725 |
|  | POY2 | 60 | 0.70 ± 0.18 | 1.4 | 0.01 | 0.727 |
|  | POY3 | 58 | 0.69 ± 0.18 | 0.0 | 0.00 | 0.727 |
|  | POY4 | 57 | 0.69 ± 0.18 | 0.0 | 0.00 | 0.808 |
|  | POY5 | 56 | 0.69 ± 0.18 | 0.0 | 0.00 | 0.860 |
|  | POY6 | 56 | 0.71 ± 0.18 | 2.9 | 0.02 | 0.459 |
|  | POY7 | 56 | 0.70 ± 0.17 | 1.4 | 0.01 | 0.685 |
|  | POY8 | 56 | 0.70 ± 0.16 | 1.4 | 0.01 | 0.651 |
| RNFL thickness (μm) | Preoperative | 62 | 70.5 ± 7.3 |  |  |  |
|  | POY1 | 61 | 71.8 ± 7.0 | 1.8 | 1.3 | 0.208 |
|  | POY2 | 60 | 74.4 ± 10.7 | 5.5 | 3.9 | 0.004* |
|  | POY3 | 58 | 74.5 ± 10.2 | 5.7 | 4.0 | 0.006* |
|  | POY4 | 57 | 74.8 ± 9.5 | 6.1 | 4.3 | 0.002* |
|  | POY5 | 56 | 74.7 ± 10.5 | 6.0 | 4.2 | 0.007* |
|  | POY6 | 56 | 74.5 ± 11.0 | 5.7 | 4.0 | 0.010* |
|  | POY7 | 56 | 75.3 ± 10.5 | 6.8 | 4.8 | 0.003* |
|  | POY8 | 56 | 74.4 ± 11.2 | 5.5 | 3.9 | 0.011* |
| GC-IPL thickness (μm) | Preoperative | 62 | 64.3 ± 6.9 |  |  |  |
|  | POY1 | 61 | 65.8 ± 6.9 | 2.3 | 1.5 | 0.052 |
|  | POY2 | 60 | 65.6 ± 10.2 | 2.0 | 1.3 | 0.268 |
|  | POY3 | 58 | 64.1 ± 9.6 | −0.3 | −0.2 | 0.904 |
|  | POY4 | 57 | 67.3 ± 9.4 | 4.7 | 3.0 | 0.059 |
|  | POY5 | 56 | 65.5 ± 10.1 | 1.9 | 1.2 | 0.326 |
|  | POY6 | 56 | 64.1 ± 10.8 | −0.3 | −0.2 | 0.989 |
|  | POY7 | 56 | 65.2 ± 9.8 | 1.4 | 0.9 | 0.452 |
|  | POY8 | 56 | 63.8 ± 10.1 | −0.8 | −0.5 | 0.791 |

*VF-MD* visual field mean deviation; *CDR* cup-to-disc ratio; *RNFL* retinal nerve fiber layer thickness; *GC-IPL* ganglion cell-inner plexiform layer; *POM* postoperative month; *POY* postoperative year

Mean ± Standard deviations are presented and statistically compared to preoperative values using Generalized Estimating Equations with sequential Bonferroni correction for multiple comparisons

Statistical significance is denoted by * for *P*<0.05 and ** for *P*<0.001
